# Isolation and genome characterization of Lloviu virus from Italian Schreibers’s bats

**DOI:** 10.1038/s41598-023-38364-7

**Published:** 2023-07-13

**Authors:** Gábor E. Tóth, Adam J. Hume, Ellen L. Suder, Safia Zeghbib, Ágota Ábrahám, Zsófia Lanszki, Zsaklin Varga, Zsófia Tauber, Fanni Földes, Brigitta Zana, Dino Scaravelli, Maria Teresa Scicluna, Andrea Pereswiet-Soltan, Tamás Görföl, Calogero Terregino, Paola De Benedictis, Isabel Garcia-Dorival, Covadonga Alonso, Ferenc Jakab, Elke Mühlberger, Stefania Leopardi, Gábor Kemenesi

**Affiliations:** 1 National Laboratory of Virology, Szentágothai Research Centre, University of Pécs, Pécs, Hungary; 2 Faculty of Sciences, Institute of Biology, University of Pécs, Pécs, Hungary; 3 Department of Virology, Immunology and Microbiology, Boston University Chobanian & Avedisian School of Medicine, Boston, MA USA; 4 National Emerging Infectious Diseases Laboratories, Boston University, Boston, MA USA; 5 Center for Emerging Infectious Diseases Policy and Research, Boston University, Boston, MA USA; 6 ST.E.R.N.A., Forlì, Department of Biological, Geological and Environmental Sciences, University of Bologna, Bologna, Italy; 7 UOC Virologia, Istituto Zooprofilattico Sperimentale del Lazio e della Toscana “M. Aleandri”, Roma, Italy; 8 Institute of Systematics and Evolution of Animals, Polish Academy of Sciences, Kraków, Poland; 9 OIE Collaborating Centre and National Reference Centre for Infectious Diseases at the Animal-Human Interface, Istituto Zooprofilattico Sperimentale delle Venezie, Legnaro, Italy; 10 INIA-CSIC, Centro Nacional Instituto Nacional de Investigación y Tecnología Agraria y Alimentaria, Madrid, Spain

**Keywords:** Virology, Viral reservoirs, Infectious diseases

## Abstract

*Lloviu cuevavirus* (LLOV) was the first identified member of *Filoviridae* family outside the *Ebola* and *Marburgvirus* genera. A massive die-off of Schreibers’s bats (*Miniopterus schreibersii*) in the Iberian Peninsula in 2002 led to its initial discovery. Recent studies with recombinant and wild-type LLOV isolates confirmed the zoonotic nature of the virus in vitro. We examined bat samples from Italy for the presence of LLOV in an area outside of the currently known distribution range of the virus. We detected one positive sample from 2020, sequenced the complete coding region of the viral genome and established an infectious isolate of the virus. In addition, we performed the first comprehensive evolutionary analysis of the virus, using the Spanish, Hungarian and the Italian sequences. The most important achievement of this study is the establishment of an additional infectious LLOV isolate from a bat sample using the SuBK12-08 cells, demonstrating that this cell line is highly susceptible to LLOV infection and confirming the previous observation that these bats are effective hosts of the virus in nature. This result further strengthens the role of bats as the natural hosts for zoonotic filoviruses.

## Introduction

Some viruses in the *Filoviridae* family can cause serious illness and mortality in humans, including Ebola and Marburg viruses^1,2^. However, some members, like the Reston virus, do not cause disease in humans^3,4^. While ebolaviruses have received significant attention from the public and health experts due to past outbreaks, including the West African Ebola outbreak in 2013–2016 and a recent outbreak in Uganda, other filoviruses also pose a potential threat^5–7^. Research has shown that certain bat species are suspected as natural reservoirs for the Ebola virus, while only the Marburg and Ravn viruses have been isolated directly from bats before the recent isolation of Lloviu virus^8–11^. The natural host for other filoviruses is still unknown, but bats are considered as the likely natural hosts for these viruses. In recent years, several new filoviruses have been described in bats from Asia^12,13^, Africa^14,15^, and Europe^16^.

*Lloviu cuevavirus* (LLOV) was the first identified member of Filoviridae family outside the Ebola and Marburgvirus genera. Widespread, consecutive die-off events of Schreibers’s bats (*Miniopterus schreibersii*) in the Iberian Peninsula in 2002 led to its discovery. Almost complete genome sequence of the virus was retrieved with various sequencing approaches, although the infectious virus was not isolated from these bats at that time^17^. It has been reported that Schreibers’s bats in Spain and Hungary are LLOV-seropositive, confirming that the virus circulates there^18,19^. Viral RNA was sequenced from Spanish and Hungarian samples, and recently, an infectious isolate was established from the blood sample of a naturally infected bat in Hungary^17,19,20^.

Studies with recombinant and wild-type LLOV isolates confirmed the susceptibility of human-derived cell lines and primary human macrophages to LLOV infection in vitro. Based on these data, LLOV is now considered as a potential zoonotic virus with unknown pathogenicity to humans and bats^19,21^.

The ability of LLOV infection to cause disease in bats is unclear. Although no causative relationship has been established between Schreibers’s bat die-off events and circulation of LLOV within bat populations to date, the correlation is noteworthy^19,22^. The recent isolation of the virus from Schreibers’s bats in Hungary confirmed the role of these bats as hosts for LLOV and perhaps as a reservoir species. It is unclear if the first detection of the virus in Spain coincided with an initial introduction of LLOV to European *M. schreibersii* populations. Schreibers’s bats roost in large colonies and are migratory, making them ideal reservoir hosts for the rapid and effective spread of certain infectious agents within their geographic distribution. Thus, it is possible that LLOV was already broadly circulating in these bats across the continent prior to the first detection of the virus in Spain in 2002.

The aim of this study is to gain a better understanding of the presence, evolution, and connection to Schreibers’s bats of LLOV in previously unexplored geographical regions by using retrospective samples.

## Results

**Figure 1:**
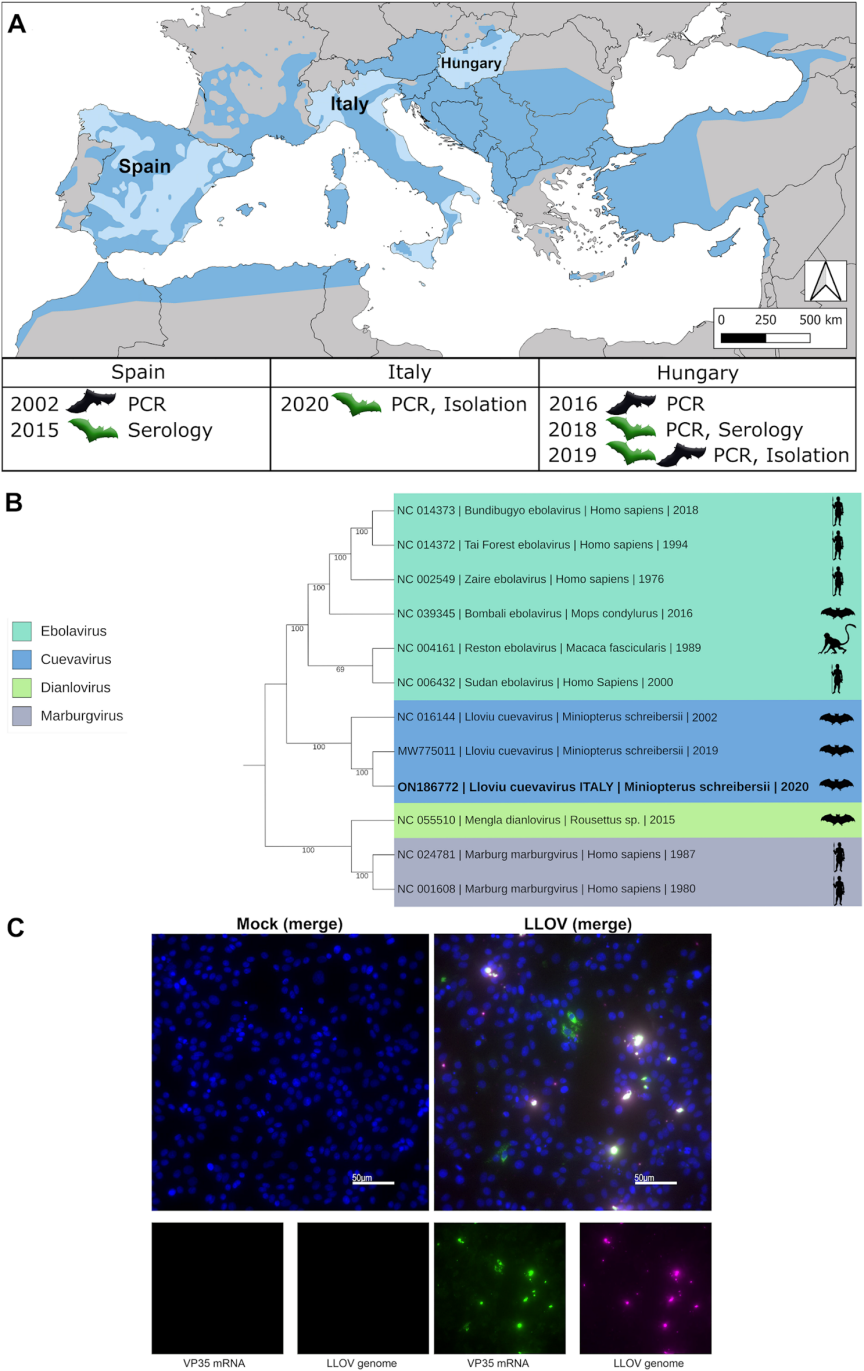
(**A**) Geographic distribution of Schreibers’s bats and identified locations of LLOV detection. The map shows the currently known distribution range of Schreibers’s bats (darker blue). Countries in which LLOV has been found are named on the map. The year and methods of detection are indicated below the countries of sample origin. Bat pictograms in normal position (green) indicate samples from live animals while upside down bats (black) indicate samples from bat carcasses. (**B**) Phylogenetic analyses of mammalian-associated filovirus reference genomes, including the available bat-derived LLOV sequences. The Maximum Likelihood tree was built using general time reversible model of substitution with gamma distributed rate variation and was tested with 1000 bootstrap replicates. Novel sequence data is highlighted with bold letters and available in the GenBank database under the accession number: ON186772 (**C**) SuBK12-08 cells were left uninfected (mock) or infected with the Italian LLOV isolate. At 1-day post-infection, cells were fixed with 10% formalin and stained by RNA FISH using probes targeting the negative sense viral genome (magenta) and the positive sense VP35 mRNA (green). Cell nuclei are stained with DAPI (blue). The map was created using the open-source software QGIS Desktop 3.24.1 (https://qgis.org/en/site/) and was modified by incorporating geographic distribution parameters of *Miniopterus* *schreibersii* sourced from the IUCN database (The IUCN Red List of Threatened Species, Version 2022–2).

To further explore the occurrence of LLOV in the wider distribution range of these bats and better understand its ecology and evolution, we screened 276 samples from Schreibers’s bats collected from four different locations in northern and central Italy between 2018 and 2021 (Fig. 1A), including 173 blood clots and 103 lung sections.

While all 103 lung samples tested negative for LLOV RNA, one of the 173 blood clot samples was positive with a Ct value of 21.07, corresponding to approximately 3.8 × 10^8^ genomic copies per mL. The sample belonged to a set of 56 blood clots collected in September 2020 from a single colony. Amplicon-based, targeted Nanopore sequencing was used to determine the complete coding sequence of the viral genome. With the combination of the three applied primer set, 615,045 reads were mapped to the reference genome which resulted in a 27,489.5 × mean coverage. The retrieved sequence was 18,861 nucleotides in length, covering all seven genes (NP, VP35, VP40, GP, VP30, VP24, and L) (GenBank: ON186772). Compared to the reference LLOV genome from Spain (GenBank: NC_016144.1) and the Hungarian LLOV sequence (GenBank: MW775011), the sequence from the Italian LLOV sample showed 99.13% and 99.86% identity at nucleotide level, respectively. The phylogenetic analysis showed that the Italian LLOV isolate is more closely related to the Hungarian isolate (Fig. 1B).

Analysis of the mutational landscape gives the first insight into the evolution of *Lloviu cuevavirus*. Comparing to the published Spanish LLOV sequence, the dN/dS ratios of each gene indicate that only GP (Hungarian: 1.38, Italian: 1.5) had a ratio above 1, implying the presence of positive selection pressure. The highest rate of synonymous mutations was found in the VP24 gene. Using the Spanish LLOV sequence as a baseline, we observed a roughly linear mutational rate for the Hungarian and Italian LLOV sequences with respect to the time of sample collection (Figure S1B). Non-synonymous mutations are summarized in Table S1. While several amino acid coding changes were found throughout the LLOV genome most of these were localized in GP (Table S1). Interestingly, some of these mutations have been previously reported and linked to virus adaptation into a new species, such in the case of amino acid positions 465 and 493 that have been found in EBOV GP when adapted to a new host^23^. Another interesting coding change we identified was in amino acid position 16 (F16L) of VP24. A similar but inverted change was found in aa position 26 (L26F) for EBOV VP24. This mutation was described as a molecular determinant of virulence in guinea pig model, with this single amino acid change in EBOV proving to be sufficient to cause increased pathogenesis and death in guinea pigs^24^. This observation was further confirmed in another study where EBOV amino acid variations associated with increased pathogenesis were studied^23^. Considering the phylogenetic relatedness and functional similarities of LLOV to EBOV, these mutations are prominent targets of functional experiments in the future to better understand LLOV evolution and pathogenesis.

In addition to sequence-based analyses we conducted in vitro isolation experiments using the LLOV-positive sample to infect the *Miniopterus*-derived cell line SuBK12-08^25,26^. After the first passage of the supernatant, cytopathic effect (CPE) was observed in the cells, similar to previous LLOV isolation studies^19^. To verify the presence of replicative LLOV, we performed RNA fluorescent in situ hybridization (FISH) staining with LLOV RNA-specific probes. We observed accumulation of LLOV genomic RNA in virus-induced inclusion bodies and a diffuse distribution of VP35 mRNA throughout the cytoplasm of the infected bat cells which are hallmarks of active viral replication (Fig. 1C). This confirms the successful isolation of the second infectious LLOV isolate from bats in Europe.

## Discussion

Lloviu virus has now been detected in three European countries, suggesting a more widespread occurrence of the virus than previously anticipated (Fig. 1A)^17–20^. Schreibers’s bats are still the only identified host for the virus, and it is conceivable that LLOV might be present throughout the entire geographic range of this bat species. Schreibers’s bat is a fast-flying regional migrant, whose migrations can greatly vary. It usually occupies roosts in a few tens of kilometres from each other but can cover distances of several hundred kilometres^27^. It has one of the largest colonies in Europe, in some countries, tens of thousands of bats roost in the same cave or mine. The animals in the colonies are usually in close contact, sometimes with other species like lesser (*Myotis blythii*) or greater mouse-eared bats (*M. myotis*). These traits and habits make ideal circumstances for the spread of diseases between and within colonies, possibly causing serious conservational threats for the bats^22^.

Considering the zoonotic potential of LLOV, there is an urgent need to better understand the evolution, ecological background, and molecular features of LLOV. Our optimised qRT-PCR system combined with a novel primer set allows for the rapid and sensitive screening for LLOV viral RNA in various samples derived from bats. Large-scale screening of archived and newly collected samples would help to investigate our hypothesis about the wide geographic distribution of the virus among these bats. We verified our hypothesis about the broader geographic range of Schreibers’s bats infected with LLOV and widened the possible active transmission area of the virus to the geographic range of its currently known host.

The high viral load in the blood sample of the single LLOV-positive bat is in accordance with recent reports on LLOV in Schreibers’s bats in Hungary and with previous observations for other filoviruses in bats^28,29^. LLOV-positive bats were detected in September both in Hungary and in Italy, although it is not yet clear if this points to a potential seasonality of the virus or if this reflects a random occurrence pattern. In case of European Bat Lyssavirus type 2, the peak season of virus prevalence correlated with the autumn swarming season of the bats^30^.

The most important achievement of this study is the isolation of an additional infectious LLOV isolate from a bat sample collected in Italy using the SuBK12-08 cells, demonstrating that this cell line is highly susceptible to LLOV infection^19^. This is consistent with a previous report which found these cells to be highly susceptible to Vesicular Stomatitis Virus pseudotyped with LLOV GP^25,26^.

Notably, there is still no evidence for actual spillover of LLOV from bats to humans, but the repeated isolation of infectious LLOV from Schreibers’s bats highlight the need for additional studies to better understand potential spillover mechanisms. Urine and faeces of LLOV-infected bats were found to be negative in a recent study^19^. Extensive surveillance and sequencing efforts are essential to understand the potential role of Schreibers’s bats in the natural circulation of this virus and its evolution. Notably, these bats are mainly considered to be cave-dwelling, although in rare cases they might respond and adapt to habitat disruption by moving closer to humans to artificial roost sites. Indeed, the recent discovery of roosts hosting hundreds of Schreibers’s bats within urban settings in Italy raises concerns about the increasing risk of both direct bat-human contact as well as indirect contact through the spillover to domestic animals^31^. This also emphasizes the importance of the conservation of bats and their habitats as a means for limiting potential zoonotic spillovers to humans^32^.

Cases of mass die-offs of Schreibers’s bats have been reported across Europe, including areas where LLOV was detected^22^. This report describes the detection of LLOV from live-sampled animals that were apparently healthy, with no reported unusual mortality events among these bats in the area. Investigating the circulation of LLOV among populations of this bat species, including the estimation and evaluation of mortality events, is crucial not only for public health but also for the conservation of Schreibers’s bats that are classified as Vulnerable on the IUCN Red List^32^.

## Materials methods

### Ethics and biosafety statement

Bats were captured during the day with hand-nets in full compliance with best practice and national and European regulations, in derogation to the Council Directive 92/43/EEC of 21 May 1992 on the conservation of natural habitats and of wild fauna and flora (authorization 38,025 of 13/08/2020 and 6831 of 15/02/2021 released by the Italian Ministry for the Ecological Transition). Animal handling was performed by experienced chiropterologists, no animals were harmed during field work. In vitro isolation procedures were performed under Biosafety Level 4 conditions within the laboratory of Szentágothai Research Centre, University of Pécs, Hungary.

All methods were carried out in accordance with relevant guidelines and regulations, detailed in and approved by the previously mentioned permissions.

### Sample collection

Bat species identification were performed according to morphological identification keys^33^. During the regular colony checking occasions in 2018 and 2019, 54 Schreibers’s bat carcasses were collected from two sampling sites in North-eastern Italy. Also, in the same region but in a different cave a bat mortality among neonates were detected and 49 additional carcasses were collected in 2020. Altogether 103 lung samples were subjected to LLOV surveillance after the necropsy. Also, 173 samples from live animals involved in this study were collected from a single colony in Central Italy. Blood samples were obtained during four active surveillance campaigns between late summer 2020 (August, September, October) and spring 2021 (April), to avoid disturbing the bat colonies during hibernation and the first month after birth. The detailed list of samples is available in the supplementary materials (S2).

### LLOV RNA detection and sequencing

Lung samples were homogenized in 300 µl Eagle’s Minimum Essential Medium (EMEM, Lonza, Switzerland) with the usage of a TissueLyser II (Qiagen, Germany). In the case of blood samples, the blood pellets were suspended in 300 µl EMEM. After a slight centrifugation (3 min – 8000 rpm), 200 µl supernatant was transferred for nucleic acid extraction.

RNA was extracted from samples using Direct-Zol RNA Miniprep kit (Zymo Research, USA) according to the manufacturer’s recommendations. For LLOV RNA detection, a novel TaqMan PCR assay was developed and optimized using the *Lloviu cuevavirus* isolate Hungary/2019/378 (GenBank: MZ541881.1). We used the following primer sequences: LLOV-Fw-scr1: 5′-AAGCATTTCCGAGTAATATGATGGTTG-3′, LLOV-Rev-scr1: 5′-TACATGGTCTCCTAGATTGCCCTG-3′, LLOV-Prob-scr1: 5′-FAM-CCTGATGAAGGAGAGTTTCTTTCTG-ZEN-3′ with the qRT-PCR Brilliant III Probe Master Mix (Agilent Technologies, USA) under these conditions: 50 °C for 10 min, 95 °C for 3 min and 50 cycles of 95 °C for 5 s, 60 °C for 30 s. All experiments were run on the MyGo Pro PCR system platform (IT-IS Life Science, Ireland). Genomic copy number per millilitre was calculated from the Ct value and standard dilutions. Previously published LLOV specific targeted amplicon sequencing was used to sequence the native positive RNA sample. In brief, cDNA was generated using SuperScript IV (Invitrogen, USA) with random hexamers. Multiplex PCR reactions were conducted with Q5 HF Polymerase (New England Biolabs, USA) using 3 different primer sets. Amplicon pools were end-repaired using NEBNext Ultra II End Repair/dA-Tailing Module (New England Biolabs, USA). Barcodes from EXP-NBD196 (Oxford Nanopore Technologies, UK) were ligated to the amplicons with NEBNext Ultra II Ligation Module (New England Biolabs, USA). For the sequencing, the LSK-110 kit (Oxford Nanopore Technologies, UK) was used, and the AMX-F motor protein was ligated to the barcoding pool with NEBNext Quick Ligation Module (New England Biolabs, USA). Cleaning steps between reactions were conducted with Ampure XP beads (Beckman Coulter, USA) using 75% ethanol except the last clean up where Small Fragment Buffer was applied. After the cleaning steps, the library was quantified using Qubit dsDNA HS Assay Kit (Invitrogen, USA) on a Qubit 4 fluorometer (Invitrogen, USA). The final library was sequenced on a R9.4.1 (FLO-MIN106D) flow cell.

### Sequence analysis

We ran the analysis under Ubuntu Linux 22.04 LTS operating system. The generated raw data was basecalled using Guppy basecaller (ONT guppy v6.3.2) with super-accurate model (dna_r9.4.1_450bps_sup config file). Demultiplexing was performed using Guppy barcoder with the necessity to find barcodes at both ends. For the quality check the NanoPlot (v1.33.0) software was used. We performed a quality (Q > 11) and length filtering with NanoFilt (v2.8.0) and 50 extra bases were trimmed^34^. The processed reads were mapped to the reference genome (NC_016144.1) using Minimap2 (v. 2.23)^35^. The SAM to BAM conversion and consensus calling was conducted with samtools and bcftools (v. 1.17)^36^. The draft sequence was polished with Medaka (v. 1.72) Complete genomes from Filoviridae family were collected and aligned using Muscle algorithm^37^. Phylogenetic analyses were conducted in MEGA 11^38^.

### In vitro isolation

SuBK12-08 cells were grown at 37 °C and 5% CO_2_ in EMEM with 1% of L-Glutamine (200 mM) (Lonza, Switzerland). The culture medium was supplemented with 2% heat inactivated Fetal Bovine Serum (FBS) (Gibco, USA), and 1% Penicillin/Streptomycin (10,000 U/mL). The positive blood clot sample was used for inoculation, after adding 200 µl serum-free EMEM. After 1 h incubation at 37 °C and 5% CO_2_, the cells were washed once with serum-free cell culture media and the final supernatant, same as cell growth supernatant was added. The cells were monitored daily for cytopathic effects (CPE). After observing CPE, supernatant was subjected to nucleic acid extraction by Direct-Zol RNA MiniPrep Kit and analyzed for the presence of LLOV RNA by RT-PCR using LLOV-specific primers.

### RNA fluorescence in situ hybridization (RNA FISH) analysis

6 × 10^5^ SubK12-08cells were seeded per well of 2-well chamber slides. One day later, the cells were mock-infected or infected with LLOV Italy. The cells were fixed 24 h post-infection in 10% formalin for at least 6 h. RNA FISH was performed using the RNAscope Multiplex Fluorescent V2 kit (Advanced Cell Diagnostics, USA) with multiplexed, custom-designed probe sets in different imaging channels specific for LLOV VP35 mRNA and negative-sense genomic RNA in the nucleoprotein (NP) gene. Probes were designed based on the reference LLOV genome sequence from Spain (Genbank: NC_016144.1). LLOV mRNA was detected in channel 1 using probes targeting the VP35 transcripts and stained with Opal 520 fluorophore (Perkin-Elmer, USA). LLOV genomic RNA was detected in channel 2 using probes targeting the negative-sense genomic sequences of the NP gene and stained with Opal 690 fluorophore (Perkin-Elmer, USA). Staining was performed according to the manufacturer’s protocol for adherent cell samples, with the exception of an additional HRP blocking step following the signal development of the probes detecting viral mRNA as per the manufacturer’s recommendation. Nuclei were stained with kit supplied DAPI following the manufacturer's protocol. Coverslips were mounted onto the chamber slides using FluorSave mounting medium, and slides were subsequently stored at 4 °C prior to imaging. Images were acquired at 40× magnification using a Nikon Ti2 Eclipse microscope and Photometrics Prime BSI camera with NIS Elements AR software.

## Supplementary Information

Supplementary Information.

## Data Availability

All data generated or analysed during this study are included in this published article and its supplementary information files. The genomic sequence data in this study have been deposited in the NCBI GenBank database under accession code: ON186772.
