# Supplementary material for: Isolation and genome characterization of Lloviu virus from Italian Schreibers’s bats
Source: Sci Rep. 2023 Jul 13;13:11310. doi: 10.1038/s41598-023-38364-7 (PMC10344946; doi:10.1038/s41598-023-38364-7)
Supplement: Supplementary file 1 — Supplementary Information. [file 41598_2023_38364_MOESM1_ESM.docx]

**Supplementary material**


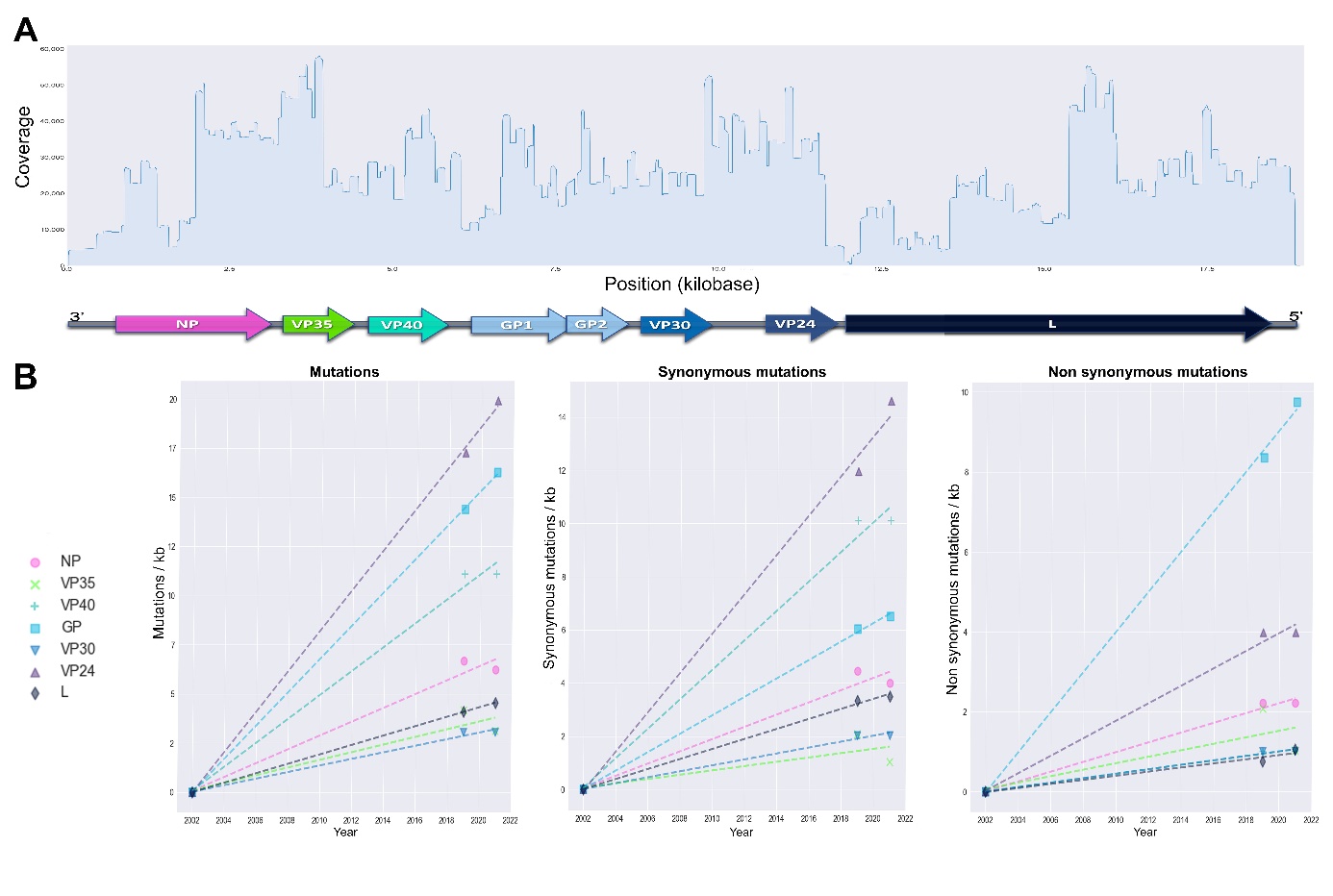


**Figure S1. (A)** The coverage data of the Italian LLOV isolate sequencing results (ON186772) with a schematic genome representation below. **(B)** The number of mutations normalized to gene length for the 7 LLOV genes for twenty years

**Table S1.** Non-synonymous mutations comparing the three currently available complete coding sequences of Lloviu virus. Reference: NC_016144, Hungarian: MW775011, Italian: ON186772.

| **Gene** | **Nucleotide position** | **aa position** | **Spanish aa position** | **Hungarian aa position** | **Italian aa position** | **Hungarian substitution per kb** | **Hungarian dN/dS ratio** | **Italian substitution per kb** | **Italian dN/dS ratio** |
| --- | --- | --- | --- | --- | --- | --- | --- | --- | --- |
| NP | 1229 | 410 | N | T | T | **2.22** | **0.50** | **2.22** | **0.56** |
| NP | 1336 | 446 | S | P | P |  |  |  |  |
| NP | 1859 | 620 | N | S | S |  |  |  |  |
| NP | 2005 | 669 | E | K | K |  |  |  |  |
| NP | 2111 | 704 | I | T | T |  |  |  |  |
| VP35 | 487 | 163 | A | T | A | **2.08** | **1.00** | **1.04** | **1.00** |
| VP35 | 622 | 208 | L | F | F |  |  |  |  |
| VP40 | 101 | 34 | P | L | L | **1.01** | **0.10** | **1.01** | **0.10** |
| GP | 493 | 165 | H | Y | Y | **8.36** | **1.38** | **9.75** | **1.50** |
| GP | 1071 | 358 | S | P | P |  |  |  |  |
| GP | 1095 | 366 | A | T | T |  |  |  |  |
| GP | 1249 | 417 | S | N | N |  |  |  |  |
| GP | 1269 | 424 | E | K | K |  |  |  |  |
| GP | 1333 | 445 | L | P | P |  |  |  |  |
| GP | 1339 | 447 | L | P | P |  |  |  |  |
| GP | 1383 | 462 | S | P | P |  |  |  |  |
| GP | 1393 | 465 | V | A | A |  |  |  |  |
| GP | 1394 | 465 | V | A | A |  |  |  |  |
| GP | 1399 | 467 | Y | H | H |  |  |  |  |
| GP | 1431 | 478 | T | P | P |  |  |  |  |
| GP | 1439 | 480 | L | P | P |  |  |  |  |
| GP | 1446 | 483 | P | S | S |  |  |  |  |
| GP | 1462 | 488 | I | I | T |  |  |  |  |
| GP | 1463 | 488 | I | I | T |  |  |  |  |
| GP | 1476 | 493 | S | P | P |  |  |  |  |
| GP | 1537 | 513 | V | A | A |  |  |  |  |
| GP | 1584 | 529 | H | H | Y |  |  |  |  |
| GP | 2058 | 687 | T | A | A |  |  |  |  |
| GP | 2076 | 693 | I | V | V |  |  |  |  |
| VP30 | 583 | 195 | A | T | T | **1.01** | **0.50** | **1.01** | **0.50** |
| VP24 | 46 | 16 | F | L | L | **3.98** | **0.33** | **3.98** | **0.27** |
| VP24 | 472 | 158 | D | N | N |  |  |  |  |
| VP24 | 554 | 185 | C | Y | Y |  |  |  |  |
| L | 237 | 79 | I | I | M | **0.76** | **0.23** | **1.06** | **0.30** |
| L | 3292 | 1098 | G | R | R |  |  |  |  |
| L | 3467 | 1156 | D | G | G |  |  |  |  |
| L | 4874 | 1625 | R | R | K |  |  |  |  |
| L | 5159 | 1720 | S | L | L |  |  |  |  |
| L | 5171 | 1724 | R | Q | Q |  |  |  |  |
| L | 5239 | 1747 | T | S | S |  |  |  |  |
